# Crystal structure, Hirshfeld surface analysis and inter­action energy and DFT studies of 3-{(2*Z*)-2-[(2,4-di­chloro­phen­yl)methyl­idene]-3-oxo-3,4-di­hydro-2*H*-1,4-benzo­thia­zin-4-yl}propane­nitrile

**DOI:** 10.1107/S2056989019005966

**Published:** 2019-05-03

**Authors:** Nada Kheira Sebbar, Brahim Hni, Tuncer Hökelek, Abdelhakim Jaouhar, Mohamed Labd Taha, Joel T. Mague, El Mokhtar Essassi

**Affiliations:** a Laboratoire de Chimie Appliquée et Environnement, Equipe de Chimie Bioorganique Appliquée, Faculté des Sciences, Université Ibn Zohr, Agadir, Morocco; b Laboratoire de Chimie Organique Hétérocyclique URAC 21, Pôle de Compétence Pharmacochimie, Av. Ibn Battouta, BP 1014, Faculté des Sciences, Université Mohammed V, Rabat, Morocco; c Department of Physics, Hacettepe University, 06800 Beytepe, Ankara, Turkey; d Department of Chemistry, Tulane University, New Orleans, LA 70118, USA

**Keywords:** crystal structure, hydrogen bond, oxygen⋯halogen inter­action, nitrile, di­hydro­benzo­thia­zine, DFT, Hirshfeld surface

## Abstract

The title compound, C_18_H_12_Cl_2_N_2_OS, consists of a di­hydro­benzo­thia­zine unit linked by a –CH group to a 2,4-di­chloro­phenyl substituent, and to a propane­nitrile unit is folded along the S⋯N axis and adopts a flattened-boat conformation. The propane­nitrile moiety is nearly perpendicular to the mean plane of the di­hydro­benzo­thia­zine unit. In the crystal, C—H_Bnz_⋯N_Prpnit_ and C—H_Prpnit_⋯O_Thz_ (Bnz = benzene, Prpnit = propane­nitrile and Thz = thia­zine) hydrogen bonds link the mol­ecules into inversion dimers, enclosing *R*_2_^2^(16) and *R*_2_^2^(12) ring motifs, which are linked into stepped ribbons extending along [110]. The ribbons are linked in pairs by complementary C=O⋯Cl inter­actions. π–π contacts between the benzene and phenyl rings, [centroid–centroid distance = 3.974 (1) Å] may further stabilize the structure. The Hirshfeld surface analysis of the crystal structure indicates that the most important contributions for the crystal packing are from H⋯H (23.4%), H⋯Cl/Cl⋯H (19.5%), H⋯C/C⋯H (13.5%), H⋯N/N⋯H (13.3%), C⋯C (10.4%) and H⋯O/O⋯H (5.1%) inter­actions. Hydrogen bonding and van der Waals inter­actions are the dominant inter­actions in the crystal packing. Computational chemistry calculations indicate that the two independent C—H_Bnz_⋯N_Prpnit_ and C—H_Prpnit_⋯O_Thz_ hydrogen bonds in the crystal impart about the same energy (*ca* 43 kJ mol^−1^). Density functional theory (DFT) optimized structures at the B3LYP/6–311 G(d,p) level are compared with the experimentally determined mol­ecular structure in the solid state. The HOMO–LUMO behaviour was elucidated to determine the energy gap.

## Abstract 2

In the title compound, the di­hydro­benzo­thia­zine moiety is folded about the S1⋯N1 axis. In the crystal, inversion dimers, generated by C—H_Bnz_⋯N_Prpnit_ (Bnz = benzene, Prpnit = propane­nitrile) hydrogen bonds, are linked into stepped ribbons extending parallel to [110] by C—H_Prpnit_⋯O_Thz_ (Thz = thia­zine) hydrogen bonds. The ribbons are joined into pairs by inversion-related C=O⋯Cl inter­actions.

## Chemical context   

1,4-Benzo­thia­zine derivatives constitute an important class of heterocyclic systems. These mol­ecules exhibit a wide range of biological applications indicating that the 1,4-benzo­thia­zine moiety is a potentially useful template in medicinal chemistry research and has therapeutic applications as anti-inflammatory (Trapani *et al.*, 1985 ▸; Gowda *et al.*, 2011 ▸), anti­pyretic (Warren & Knaus, 1987 ▸), anti-microbial (Armenise *et al.*, 2012 ▸; Rathore & Kumar, 2006 ▸; Sabatini *et al.*, 2008 ▸), anti-viral (Malagu *et al.*, 1998 ▸), anti-cancer (Gupta *et al.*, 1985 ▸; Gupta & Gupta, 1991 ▸) and anti-oxidant (Zia-ur-Rehman *et al.*, 2009 ▸) agents. 1,4-Benzo­thia­zine derivatives have also been reported as precursors for the syntheses of new compounds (Sebbar *et al.*, 2015*a* ▸; Vidal *et al.*, 2006 ▸) possessing anti-diabetic (Tawada *et al.*, 1990 ▸) and anti-corrosion activities (Ellouz *et al.*, 2016*a* ▸,*b* ▸; Sebbar *et al.*, 2016*a* ▸). They also possess biological properties (Hni *et al.*, 2019*a* ▸; Saber *et al.*, 2018 ▸; Ellouz *et al.*, 2017*a* ▸,*b* ▸, 2018 ▸; Sebbar *et al.*, 2017 ▸). As a continuation of our research work on the development of N-substituted 1,4-benzo­thia­zine derivatives and the evaluation of their potential pharmacological activities, we report herein the synthesis and the mol­ecular and crystal structures of the title compound along with the Hirshfeld surface analysis and the inter­molecular inter­action energies and the density functional theory (DFT) computational calculations carried out at the B3LYP/6–31 G(d,p) and B3LYP/6–311 G(d,p) levels, respectively.
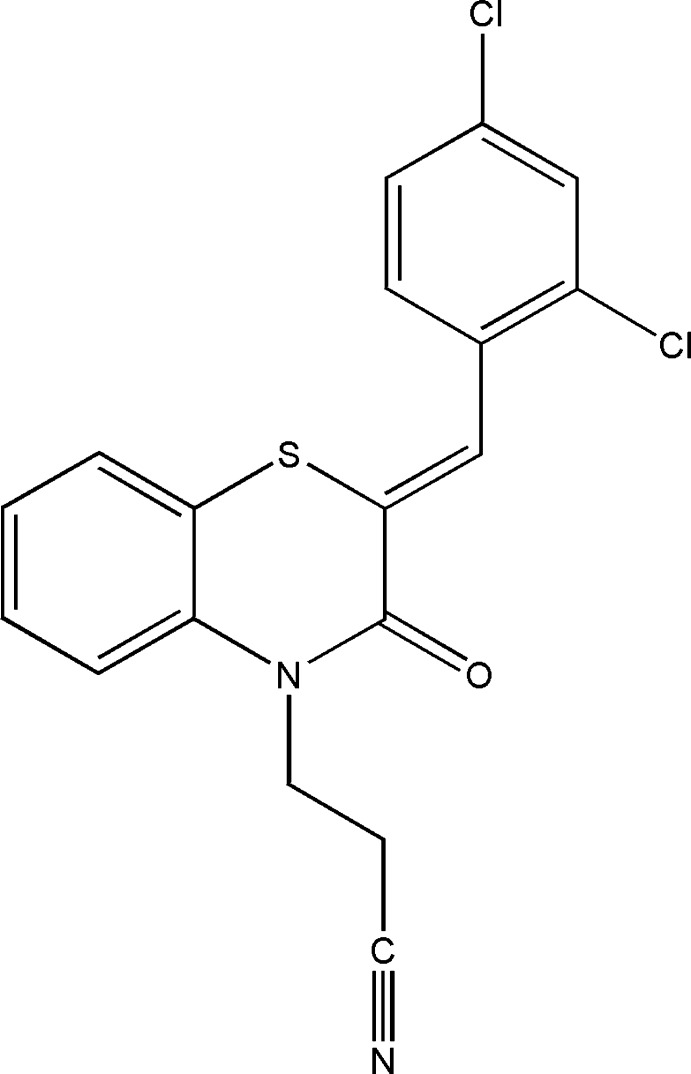


## Structural commentary   

**Figure 1:**
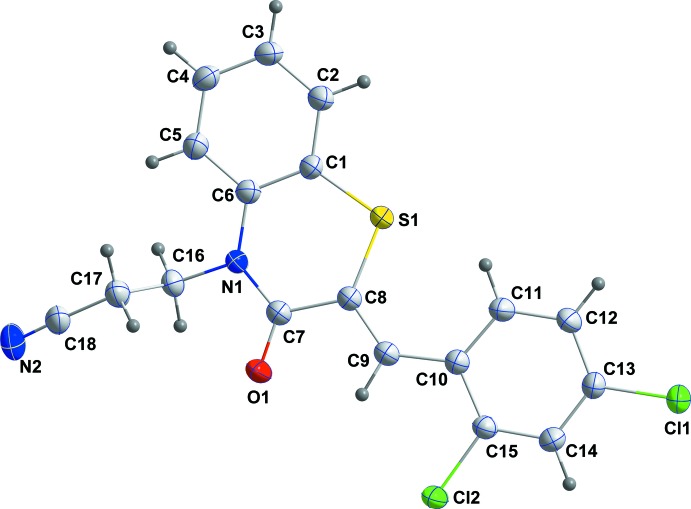
The mol­ecular structure of the title compound with the atom-numbering scheme. Displacement ellipsoids are drawn at the 50% probability level.

The title compound, (I), consists of a di­hydro­benzo­thia­zine unit linked by a –CH group to a 2,4-di­chloro­phenyl substituent and to a propane­nitrile moiety (Fig. 1 ▸). The di­hydro­benzo­thia­zine unit is folded along the S⋯N axis by 13.50 (9)°. The benzene ring, *A* (C1–C6), is oriented at a dihedral angle of 1.89 (6)° with respect to the phenyl ring, *C* (C10–C15). A puckering analysis of the heterocyclic ring *B* (S1/N1/C1/C6–C8) of the di­hydro­benzo­thia­zine unit gave the parameters *Q*_T_ = 0.1983 (15) Å, *q*_2_ = 0.1957 (17) Å, *q*_3_ = 0.0323 (19) Å, φ = 354.6 (6)° and θ = 80.8 (5)°, indicating it adopts a flattened-boat conformation. The propane­nitrile moiety is essentially perpendicular to the di­hydro­benzo­thia­zine unit, as indicated by the C7—N1—C16—C17 torsion angle of 88.6 (2)°. In heterocyclic ring *B*, the C1—S1—C8 [103.69 (9)°], S1—C8—C7 [121.12 (14)°], C8—C7—N1 [120.59 (17)°], C7—N1—C6 [126.27 (16)°], C6—C1—S1 [123.84 (15)°] and N1—C6—C1 [121.46 (17)°] bond angles are enlarged when compared with the corresponding values in the closely related compounds, (2*Z*)-2-(4-chloro­benzyl­idene)-4-[2-(2-oxooxazoliden-3-yl) eth­yl]-3,4-di­hydro-2*H*-1,4-benzo­thia­zin-3-one, (II), (Ellouz *et al.*, 2017*a* ▸) and (2*Z*)-2-[(4-fluoro­benzyl­idene]-4-(prop-2-yn-1-yl)-3,4 -di­hydro-2*H*-1,4-benzo­thia­zin-3-one, (III), (Hni *et al.*, 2019*a* ▸), and are nearly the same as the corresponding values in (2*Z*)-4-[2-(2-oxo-1,3-oxazolidin-3-yl)eth­yl]-2(phenyl­methyl­idene)-3,4-di­hydro-2*H*-1,4-benzo­thia­zin-3-one, (IV), (Sebbar *et al.*, 2016*b* ▸) and (2*Z*)-2-[(2,4-di­chloro­phen­yl)methyl­idene]-4-[2-(2-oxo-1,3-oxazolidin-3-yl)eth­yl]3,4-di­hydro-2*H*-1,4-benzo­thia­zin-3-one, (V), (Hni *et al.*, 2019*b* ▸), where the heterocyclic portions of the di­hydro­benzo­thia­zine units are planar in (IV) and non-planar in (II), (III) and (V).

## Supra­molecular features   

**Figure 2:**
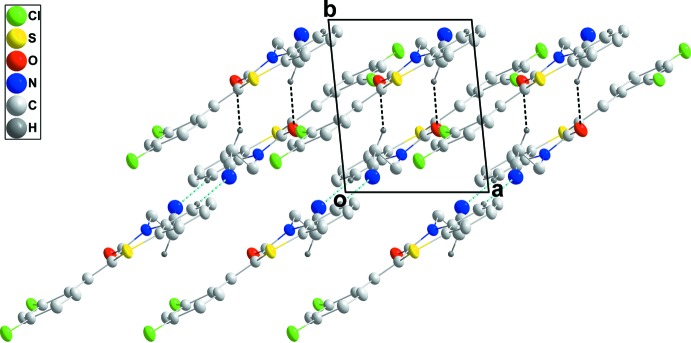
A partial packing diagram viewed along the *c*-axis direction with the C—H⋯O and C—H⋯N hydrogen bonds shown, respectively, as black and blue dashed lines.

**Figure 3:**
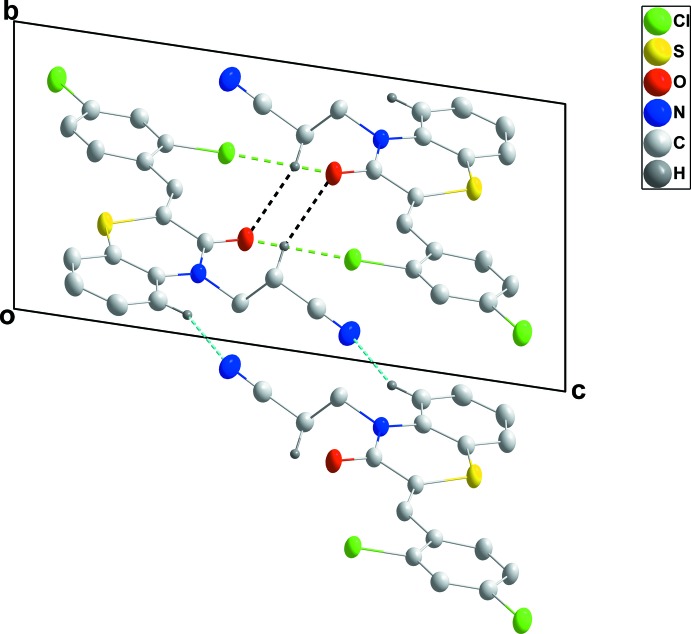
A partial packing diagram viewed along the *a*-axis direction with hydrogen bonds depicted as in Fig. 2 ▸, and C=O⋯Cl inter­actions as green dashed lines.

In the crystal, inversion dimers are formed by C—H_Bnz_⋯N_Prpnit_ (Bnz = benzene and Prpnit = propane­nitrile) hydrogen bonds (Table 1 ▸ and Fig. 2 ▸), enclosing 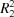
(16) ring motifs, and these units are linked into stepped ribbons extending along [110] by inversion-related C—H_Prpnit_⋯O_Thz_ (Thz = thia­zine) hydrogen bonds (Table 1 ▸ and Fig. 2 ▸), enclosing 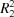
(12) ring motifs. The ribbons are arranged in pairs with inversion-related Cl2⋯O1 contacts of 3.027 (2) Å and C15=O1⋯Cl2 angles of 170.41 (7)° (Fig. 3 ▸). The contact is noticeably less than the sum of the van der Waals radii (3.27 Å), and the contact and angle compare well with corresponding parameters found in the structure of 2,5-di­chloro-1,4-benzo­quinone and attributed to attractive O⋯Cl inter­actions (Lommerse *et al.*, 1996) ▸. The π–π contacts between the benzene (C1–C6, centroid *Cg*1) and 2,4-dichlorophenyl rings (C10–C15, centroid *Cg*3) [*Cg*1⋯*Cg*3(*x* − 1, *y* − 1, *z*) = 3.974 (1) Å] may further stabilize the structure.

## Hirshfeld surface analysis   

**Figure 4:**
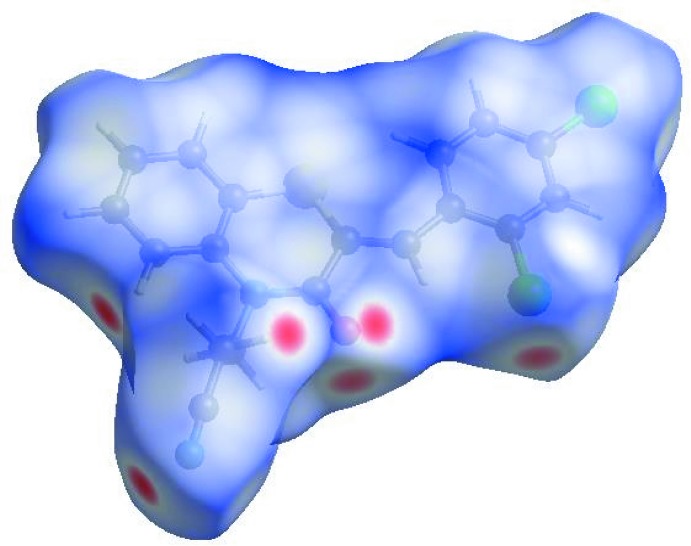
View of the three-dimensional Hirshfeld surface of the title compound plotted over *d*_norm_ in the range −0.2386 to 1.2893 a.u.

**Figure 5:**
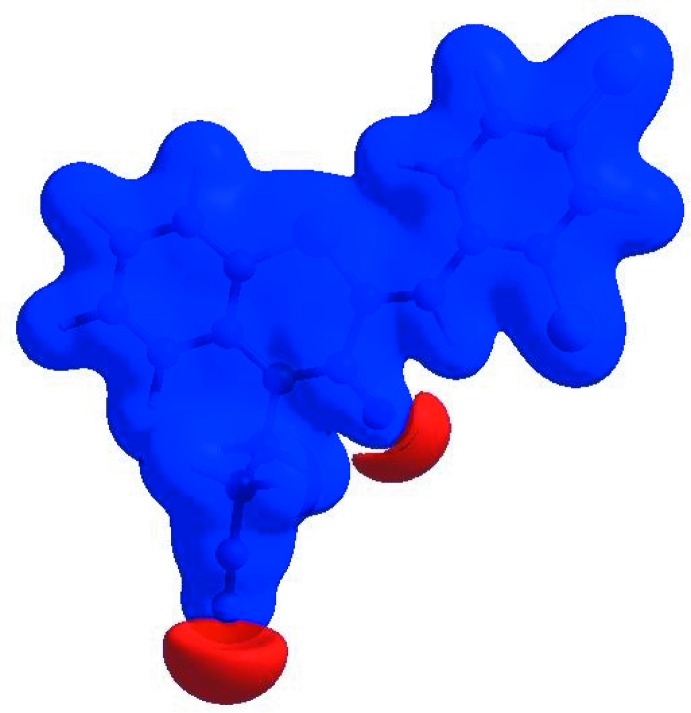
View of the three-dimensional Hirshfeld surface of the title compound plotted over electrostatic potential energy in the range −0.0500 to 0.0500 a.u. using the STO-3 G basis set at the Hartree–Fock level of theory. Hydrogen-bond donors and acceptors are shown as blue and red regions around the atoms corresponding to positive and negative potentials, respectively.

**Figure 6:**
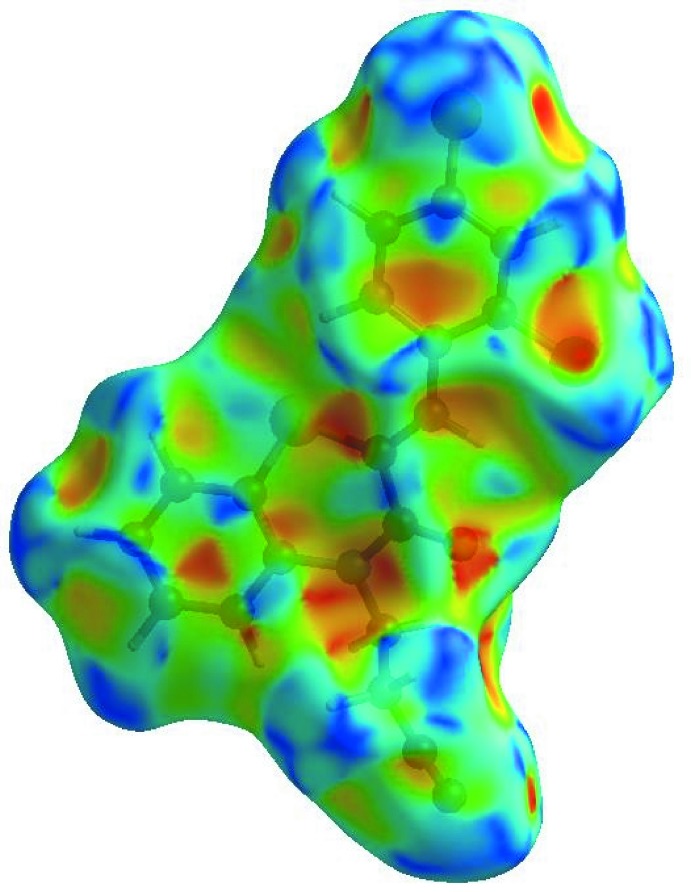
Hirshfeld surface of the title compound plotted over shape-index.

In order to visualize the inter­molecular inter­actions in the crystal of the title compound, a Hirshfeld surface (HS) analysis (Hirshfeld, 1977 ▸; Spackman & Jayatilaka, 2009 ▸) was carried out by using *CrystalExplorer17.5* (Turner *et al.*, 2017 ▸. In the HS plotted over *d*_norm_ (Fig. 4 ▸), the white surface indicates contacts with distances equal to the sum of van der Waals radii, and the red and blue colours indicate distances shorter (in close contact) or longer (distinct contact) than the van der Waals radii, respectively (Venkatesan *et al.*, 2016 ▸). The bright-red spots indicate their roles as the respective donors and/or acceptors; they also appear as blue and red regions corresponding to positive and negative potentials on the HS mapped over electrostatic potential (Spackman *et al.*, 2008 ▸; Jayatilaka *et al.*, 2005 ▸) shown in Fig. 5 ▸. The blue regions indicate positive electrostatic potential (hydrogen-bond donors), while the red regions indicate negative electrostatic potential (hydrogen-bond acceptors). The shape-index of the HS is a tool to visualize the π–π stacking by the presence of adjacent red and blue triangles; if there are no adjacent red and/or blue triangles, then there are no π–π inter­actions. Fig. 6 ▸ clearly suggest that there are π–π inter­actions in (I).

**Figure 7:**
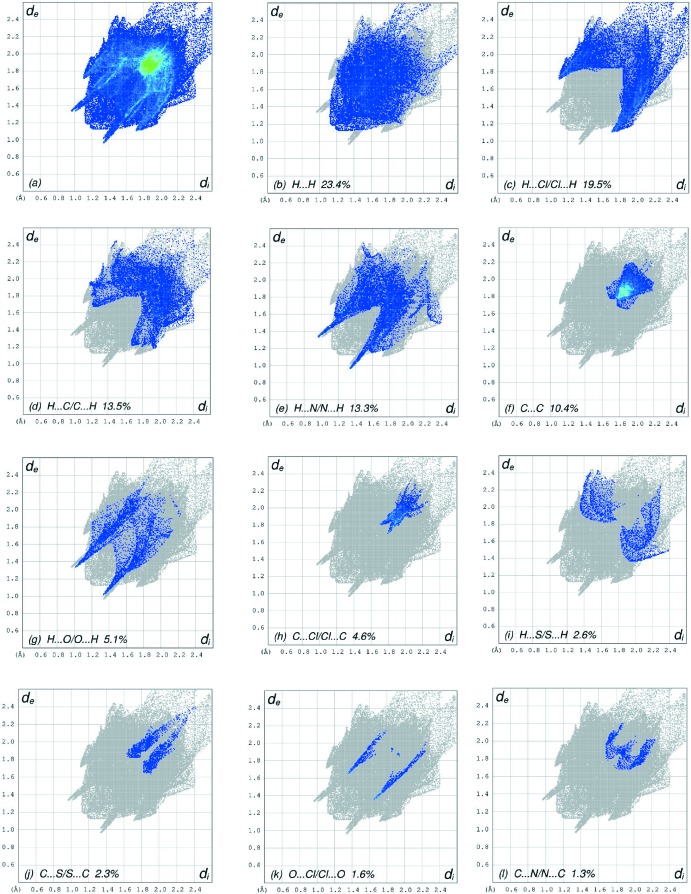
The full two-dimensional fingerprint plots for the title compound, showing (*a*) all inter­actions, and delineated into (*b*) H⋯H, (*c*) H⋯Cl/Cl⋯H, (*d*) H⋯C/C⋯H, (*e*) H⋯N/N⋯H, (*f*) C⋯C, (*g*) H⋯O/O⋯H, (*h*) C⋯Cl/Cl⋯C, (i) H⋯S/S⋯H, (*j*) C⋯S/S⋯C, (*k*) O⋯Cl/Cl⋯O and (*l*) C⋯N/N⋯C inter­actions. The *d*_i_ and *d*_e_ values are the closest inter­nal and external distances (in Å) from given points on the Hirshfeld surface contacts.

The overall two-dimensional fingerprint plot, Fig. 7 ▸ *a*, and those delineated into H⋯H, H⋯Cl/Cl⋯H, H⋯C/C⋯H, H⋯N/N⋯H, C⋯C, H⋯O/O⋯H, C⋯Cl/Cl⋯C, H⋯S/S⋯H, C⋯S/S⋯C, O⋯Cl/Cl⋯O and C⋯N/N⋯C contacts (McKinnon *et al.*, 2007 ▸) are illustrated in Fig. 7 ▸ *b*–*l*, respectively, together with their relative contributions to the Hirshfeld surface. The most important inter­action is H⋯H (Table 2 ▸), contributing 23.4% to the overall crystal packing, which is reflected in Fig. 7 ▸ *b* as widely scattered points of high density due to the large hydrogen content of the mol­ecule with the small split tips at *d*_e_ + *d*_i_ = 2.32 Å. The pair of wings in the fingerprint plot delineated into H⋯Cl/Cl⋯H contacts (19.5% contribution) have a nearly symmetrical distribution of points, Fig. 7 ▸ *c*, with the thin edges at *d*_e_ + *d*_i_ = 2.82 Å. In the absence of C—H⋯π inter­actions, the wings in the fingerprint plot delineated into H⋯C/C⋯H contacts (13.5%) also have a nearly symmetrical distribution of points, Fig. 7 ▸ *d*, with the thick edges at *d*_e_ + *d*_i_ ∼2.90 Å. The wings in the fingerprint plot delineated into H⋯N/N⋯H contacts (13.3%, Fig. 7 ▸ *e*) have as pair of spikes with the tips at *d*_e_ + *d*_i_ = 2.30 Å. The C⋯C contacts (10.4%, Fig. 7 ▸ *f*) have an arrow-shaped distribution of points with the tip at *d*_e_ = *d*_i_ ∼1.78 Å. The H⋯O/O⋯H (5.1%, Fig. 7 ▸ *g*) and C⋯Cl/Cl⋯C (4.6%, Fig. 7 ▸ *h*) contacts (Table 2 ▸) are viewed as pairs of thin spikes with the tips at *d*_e_ + *d*_i_ = 2.34 and 3.50 Å, respectively. Finally, the H⋯S/S ⋯ H (2.6%, Fig. 7 ▸ *i*) and C⋯S/S⋯C (2.3%, Fig. 7 ▸ *j*) contacts are seen as pairs of wide spikes with the tips at *d*_e_ + *d*_i_ ∼3.30 and 3.48 Å, respectively.

**Figure 8:**
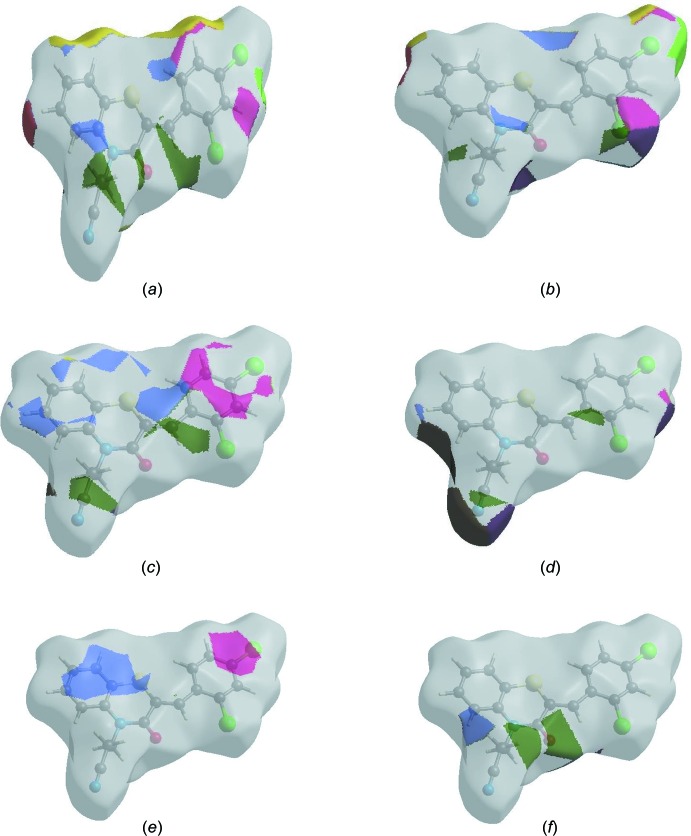
The Hirshfeld surface representations with the function *d*_norm_ plotted onto the surface for (*a*) H⋯H, (*b*) H⋯Cl/Cl⋯H, (*c*) H⋯C/C⋯H, (*d*) H⋯N/N⋯H, (*e*) C⋯C and (*f*) H⋯O/O⋯H inter­actions.

The Hirshfeld surface representations with the function *d*_norm_ plotted onto the surface are shown for the H⋯H, H⋯Cl/Cl⋯H, H⋯C/C⋯H, H ⋯ N/N⋯H, C⋯C and H⋯O/O⋯H inter­actions in Fig. 8 ▸ *a*–*f*, respectively.

The Hirshfeld surface analysis confirms the importance of H-atom contacts in establishing the packing. The large number of H⋯H, H⋯Cl/Cl⋯H, H ⋯ C/C⋯H and H⋯N/N⋯H inter­actions suggest that van der Waals inter­actions and hydrogen bonding play the major roles in the crystal packing (Hathwar *et al.*, 2015 ▸).

## Inter­action energy calculations   

The inter­molecular inter­action energies were calculated using the CE–B3LYP/6–31G(d,p) energy model available in *CrystalExplorer17.5* (Turner *et al.*, 2017 ▸), where a cluster of mol­ecules is generated by applying crystallographic symmetry operations with respect to a selected central mol­ecule within a default radius of 3.8 Å (Turner *et al.*, 2014 ▸). The total inter­molecular energy (*E*_tot_) is the sum of electrostatic (*E*_ele_), polarization (*E*_pol_), dispersion (*E*_dis_) and exchange-repulsion (*E*_rep_) energies (Turner *et al.*, 2015 ▸) with scale factors of 1.057, 0.740, 0.871 and 0.618, respectively (Mackenzie *et al.*, 2017 ▸). Hydrogen-bonding inter­action energies (in kJ mol^−1^) were calculated to be −13.0 (*E*_ele_), −1.8 (*E*_pol_), −68.0 (*E*_dis_), 48.3 (*E*_rep_) and −44.4 (*E*_tot_) for the C—H_Bnz_⋯N_Prpnit_ hydrogen-bonding inter­action and −37.3 (*E*_ele_), −9.3 (*E*_pol_), −19.0 (*E*_dis_), 33.7 (*E*_rep_) and −42.0 (*E*_tot_) for C—H_Prpnit_⋯O_Thz_.

## DFT calculations   

**Figure 9:**
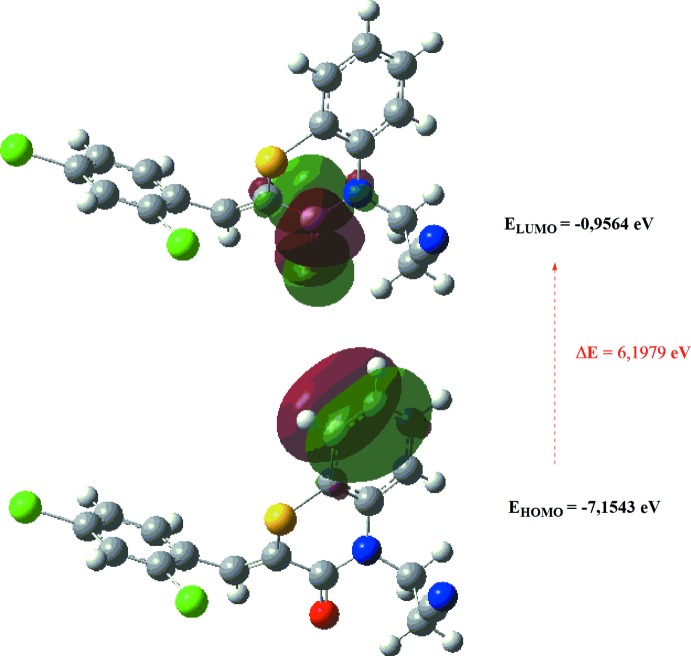
The energy band gap of the title compound.

The optimized structure of the title compound in the gas phase was generated theoretically *via* density functional theory (DFT) using standard B3LYP functional and 6–311 G(d,p) basis-set calculations (Becke, 1993 ▸) as implemented in *GAUSSIAN 09* (Frisch *et al.*, 2009 ▸). The theoretical and experimental results were in good agreement. The highest-occupied mol­ecular orbital (HOMO), acting as an electron donor, and the lowest-unoccupied mol­ecular orbital (LUMO), acting as an electron acceptor, are very important parameters for quantum chemistry. When the energy gap is small, the mol­ecule is highly polarizable and has high chemical reactivity. The electron transition from the HOMO to the LUMO energy level is shown in Fig. 9 ▸. The HOMO and LUMO are localized in the plane extending from the whole 3-[(2*Z*)-2-[(2,4-di­chloro­phen­yl)methyl­idene]-3-oxo-3,4-di­hydro-2*H*-1,4-benzo­thia­zin-4-yl]propane­nitrile ring. The energy band gap [Δ*E* = *E*_LUMO_ − *E*_HOMO_] of the mol­ecule is about 6.1979 eV, and the frontier mol­ecular orbital energies, *E*_HOMO_ and *E*_LUMO_ are −7.1543 and −0.9564 eV, respectively.

## Database survey   

A search in the Cambridge Structural Database (Groom *et al.*, 2016 ▸; updated to March 2019), for compounds containing the fragment II (*R*_1_ = Ph, *R*_2_ = C), gave 14 hits. With *R*_1_ = Ph and *R*_2_ = CH_2_C≡CH **IIa** (Sebbar *et al.*, 2014*a* ▸), CH_2_COOH **IIb** (Sebbar *et al.*, 2016*c* ▸), **IIc** (Sebbar *et al.*, 2016*b* ▸) and **IIf** (Sebbar *et al.*, 2015*b* ▸), there are other examples with *R*_1_ = 4-FC_6_H_4_ and *R*_2_ = CH_2_C≡CH **IIa** (Hni *et al.*, 2019*a* ▸), *R*_1_ = 4-ClC_6_H_4_ and *R*_2_ = CH_2_Ph2 **IId** (Ellouz *et al.*, 2016*c* ▸) and *R*_1_ = 2-ClC_6_H_4_, *R*_2_ = CH_2_C≡CH **IIa** (Sebbar *et al.*, 2017 ▸). In all these compounds, the configuration about the benzyl­idene C=CHC_6_H_5_ bond is *Z*, and in the majority of these, the heterocyclic ring is quite non-planar with the dihedral angle between the plane defined by the benzene ring plus the nitro­gen and sulfur atoms and that defined by nitro­gen and sulfur and the other two carbon atoms separating them ranging from *ca* 29° (**IIa**) to 36° (**IIf**). The other three (**IIa**, **IIc**) have the benzo­thia­zine unit nearly planar with a corresponding dihedral angle of *ca* 3–4°
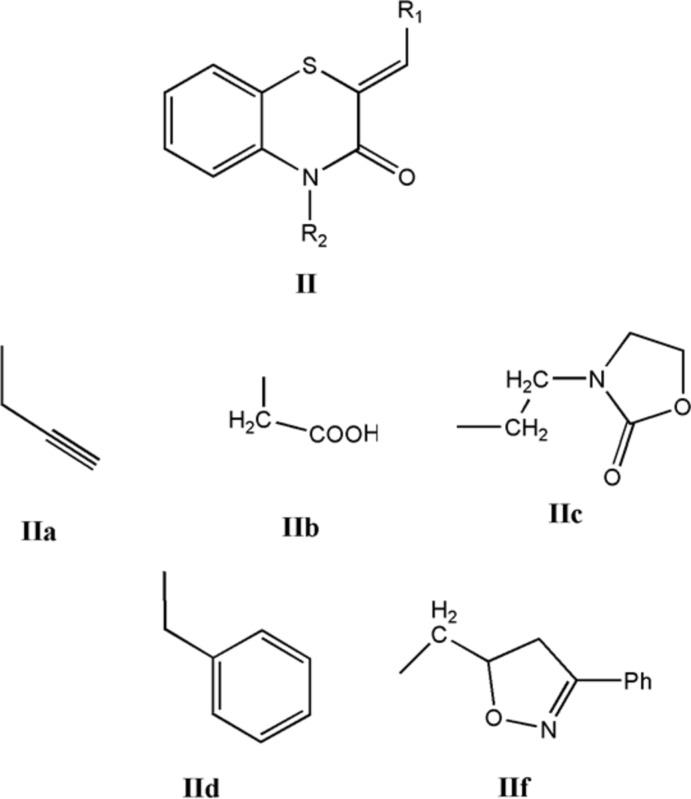
.

## Synthesis and crystallization   

3-Bromo­propane­nitrile (2.0 mmol) was added to a mixture of (*Z*)-2-(2,4-di­chloro­benzyl­idene)-2*H*-1,4-benzo­thia­zin-3(4*H*)-one (1.8 mmol), potassium carbonate (2.0 mmol) and tetra *n*-butyl ammonium bromide (0.15 mmol) in DMF (20 ml). Stirring was continued at room temperature for 12 h. The salts were removed by filtration and the filtrate was concentrated under reduced pressure. The residue was separated by chromatography on a column of silica gel with ethyl acetate–hexane (1/9) as eluent. The solid product obtained was recrystallized from ethanol to afford colourless crystals (yield: 82%).

## Refinement   

Crystal data, data collection and structure refinement details are summarized in Table 3 ▸. C-bound H atoms were positioned geometrically (C—H = 0.95 Å for aromatic and methine H atoms and 0.99 Å for methyl­ene H atoms) and constrained to ride on their parent atoms, with *U*_iso_(H) = 1.2*U*_eq_(C).

## Supplementary Material

Crystal structure: contains datablock(s) I, global. DOI: 10.1107/S2056989019005966/lh5901sup1.cif

Structure factors: contains datablock(s) I. DOI: 10.1107/S2056989019005966/lh5901Isup2.hkl

Supporting information file. DOI: 10.1107/S2056989019005966/lh5901Isup3.cdx

CCDC reference: 1913051

Additional supporting information:  crystallographic information; 3D view; checkCIF report

## Figures and Tables

**Table 1:** Hydrogen-bond geometry (Å, °)

*D*—H⋯*A*	*D*—H	H⋯*A*	*D*⋯*A*	*D*—H⋯*A*
C5—H5⋯N2^viii^	0.95	2.43	3.282 (3)	149
C17—H17*A*⋯O1^vii^	0.99	2.45	3.337 (3)	149

Symmetry codes: (vii) 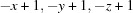; (viii) 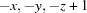.

**Table 2:** Selected interatomic distances (Å)

Cl2⋯C18^i^	3.649 (2)	N2⋯H16*A* ^ix^	2.81
Cl2⋯C7^ii^	3.520 (2)	C2⋯C11^vi^	3.569 (3)
Cl2⋯O1^i^	3.0269 (15)	C4⋯C12^x^	3.577 (3)
Cl1⋯H2^iii^	3.00	C4⋯C8^vi^	3.490 (3)
Cl1⋯H4^iv^	2.94	C5⋯C14^x^	3.557 (3)
Cl2⋯H17*A* ^ii^	3.06	C5⋯C17	3.352 (3)
Cl2⋯H9	2.51	C8⋯C4^ii^	3.490 (3)
Cl2⋯H16*B* ^v^	2.96	C9⋯C18^vii^	3.497 (3)
S1⋯N1	3.1168 (17)	C11⋯C2^ii^	3.569 (3)
S1⋯C3^ii^	3.598 (2)	C12⋯C4^v^	3.577 (3)
S1⋯C4^ii^	3.510 (2)	C14⋯C5^v^	3.557 (3)
S1⋯C11	3.162 (2)	C17⋯C5	3.352 (3)
S1⋯C14^vi^	3.578 (2)	C18⋯C9^vii^	3.497 (3)
S1⋯H11	2.47	C5⋯H16*B*	2.53
O1⋯C17	3.210 (2)	C5⋯H17*B*	2.86
O1⋯Cl2^i^	3.0269 (15)	C8⋯H11	2.94
O1⋯C17^vii^	3.336 (3)	C16⋯H5	2.48
O1⋯H17*A*	2.79	C17⋯H5	2.79
O1⋯H9	2.24	C18⋯H9^vii^	2.98
O1⋯H16*A*	2.29	H2⋯H12^xi^	2.49
O1⋯H17*A* ^vii^	2.45	H5⋯H16*B*	2.03
N2⋯C5^viii^	3.282 (3)	H5⋯H17*B*	2.26
N2⋯H5^viii^	2.43		

Symmetry codes: (i) 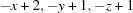; (ii) 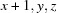; (iii) 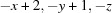; (iv) 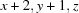; (v) 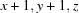; (vi) 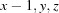; (vii) 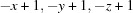; (viii) 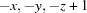; (ix) 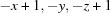; (x) 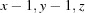; (xi) 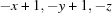.

**Table 3:** Experimental details

Crystal data	
Chemical formula	C_18_H_12_Cl_2_N_2_OS
*M*_r_	375.26
Crystal system, space group	Triclinic, *P* 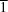
Temperature (K)	150
*a*, *b*, *c* (Å)	6.5687 (6), 7.9971 (7), 15.4939 (13)
α, β, γ (°)	98.105 (4), 94.316 (4), 95.002 (4)
*V* (Å^3^)	799.54 (12)
*Z*	2
Radiation type	Cu *K*α
μ (mm^−1^)	4.93
Crystal size (mm)	0.20 × 0.14 × 0.10

Data collection	
Diffractometer	Bruker D8 VENTURE PHOTON 100 CMOS
Absorption correction	Numerical (*SADABS*; Krause *et al.*, 2015 ▸)
*T*_min_, *T*_max_	0.47, 0.65
No. of measured, independent and observed [*I* > 2σ(*I*)] reflections	6131, 2978, 2744
*R*_int_	0.030
(sin θ/λ)_max_ (Å^−1^)	0.618

Refinement	
*R*[*F* ^2^ > 2σ(*F* ^2^)], *wR*(*F* ^2^), *S*	0.035, 0.095, 1.06
No. of reflections	2978
No. of parameters	217
H-atom treatment	H-atom parameters constrained
Δρ_max_, Δρ_min_ (e Å^−3^)	0.25, −0.35

Computer programs: *SAINT* (Bruker, 2016 ▸), *SHELXT* (Sheldrick, 2015*a* ▸), *SHELXL2018* (Sheldrick, 2015*b* ▸), *DIAMOND* (Brandenburg & Putz, 2012 ▸) and *SHELXTL* (Sheldrick, 2008 ▸).

## supplementary crystallographic information

### Crystal data

**Table:**

C_18_H_12_Cl_2_N_2_OS	*Z* = 2
*M**_r_* = 375.26	*F*(000) = 384
Triclinic, *P*1	*D*_x_ = 1.559 Mg m^−^^3^
*a* = 6.5687 (6) Å	Cu *K*α radiation, λ = 1.54178 Å
*b* = 7.9971 (7) Å	Cell parameters from 5359 reflections
*c* = 15.4939 (13) Å	θ = 5.6–72.4°
α = 98.105 (4)°	µ = 4.93 mm^−^^1^
β = 94.316 (4)°	*T* = 150 K
γ = 95.002 (4)°	Block, light yellow
*V* = 799.54 (12) Å^3^	0.20 × 0.14 × 0.10 mm

### Data collection

**Table:**

Bruker D8 VENTURE PHOTON 100 CMOS diffractometer	2978 independent reflections
Radiation source: INCOATEC IµS micro-focus source	2744 reflections with *I* > 2σ(*I*)
Mirror monochromator	*R*_int_ = 0.030
Detector resolution: 10.4167 pixels mm^-1^	θ_max_ = 72.4°, θ_min_ = 5.6°
ω scans	*h* = −8→8
Absorption correction: numerical (*SADABS*; Krause *et al.*, 2015)	*k* = −9→9
*T*_min_ = 0.47, *T*_max_ = 0.65	*l* = −18→19
6131 measured reflections	

### Refinement

**Table:**

Refinement on *F*^2^	Primary atom site location: structure-invariant direct methods
Least-squares matrix: full	Secondary atom site location: difference Fourier map
*R*[*F*^2^ > 2σ(*F*^2^)] = 0.035	Hydrogen site location: inferred from neighbouring sites
*wR*(*F*^2^) = 0.095	H-atom parameters constrained
*S* = 1.06	*w* = 1/[σ^2^(*F*_o_^2^) + (0.0424*P*)^2^ + 0.4609*P*] where *P* = (*F*_o_^2^ + 2*F*_c_^2^)/3
2978 reflections	(Δ/σ)_max_ < 0.001
217 parameters	Δρ_max_ = 0.25 e Å^−^^3^
0 restraints	Δρ_min_ = −0.35 e Å^−^^3^

### Special details

**Table:**

Geometry. All esds (except the esd in the dihedral angle between two l.s. planes) are estimated using the full covariance matrix. The cell esds are taken into account individually in the estimation of esds in distances, angles and torsion angles; correlations between esds in cell parameters are only used when they are defined by crystal symmetry. An approximate (isotropic) treatment of cell esds is used for estimating esds involving l.s. planes.
Refinement. Refinement of F^2^ against ALL reflections. The weighted R-factor wR and goodness of fit S are based on F^2^, conventional R-factors R are based on F, with F set to zero for negative F^2^. The threshold expression of F^2^ > 2sigma(F^2^) is used only for calculating R-factors(gt) etc. and is not relevant to the choice of reflections for refinement. R-factors based on F^2^ are statistically about twice as large as those based on F, and R- factors based on ALL data will be even larger. H-atoms attached to carbon were placed in calculated positions (C—H = 0.95 - 0.99 Å) and included as riding contributions with isotropic displacement parameters 1.2 - 1.5 times those of the attached atoms.

### Fractional atomic coordinates and isotropic or equivalent isotropic displacement parameters (Å^2^)

**Table:**

	*x*	*y*	*z*	*U*_iso_*/*U*_eq_	
Cl1	1.46628 (8)	0.81960 (7)	0.07781 (3)	0.04020 (16)	
Cl2	1.26553 (7)	0.65190 (6)	0.38541 (3)	0.03278 (15)	
S1	0.56343 (7)	0.34226 (7)	0.16525 (3)	0.03124 (15)	
O1	0.6855 (2)	0.3626 (2)	0.42004 (9)	0.0366 (4)	
N1	0.4171 (2)	0.2189 (2)	0.33447 (10)	0.0271 (3)	
N2	0.1980 (3)	0.0890 (3)	0.60834 (14)	0.0469 (5)	
C1	0.3267 (3)	0.2389 (2)	0.17968 (13)	0.0267 (4)	
C2	0.1860 (3)	0.2031 (3)	0.10570 (14)	0.0329 (4)	
H2	0.221285	0.238965	0.052383	0.039*	
C3	−0.0034 (3)	0.1162 (3)	0.10908 (14)	0.0354 (5)	
H3	−0.097626	0.090518	0.058325	0.042*	
C4	−0.0542 (3)	0.0669 (3)	0.18748 (15)	0.0346 (5)	
H4	−0.184723	0.007778	0.190526	0.042*	
C5	0.0823 (3)	0.1028 (3)	0.26120 (14)	0.0321 (4)	
H5	0.043934	0.069093	0.314587	0.039*	
C6	0.2759 (3)	0.1878 (2)	0.25857 (13)	0.0267 (4)	
C7	0.5932 (3)	0.3289 (3)	0.34700 (13)	0.0279 (4)	
C8	0.6774 (3)	0.4023 (2)	0.27150 (13)	0.0263 (4)	
C9	0.8579 (3)	0.5003 (2)	0.29147 (13)	0.0281 (4)	
H9	0.901779	0.520563	0.352193	0.034*	
C10	0.9972 (3)	0.5804 (2)	0.23760 (13)	0.0269 (4)	
C11	0.9552 (3)	0.5907 (3)	0.14823 (14)	0.0331 (4)	
H11	0.824104	0.546039	0.120352	0.040*	
C12	1.0964 (3)	0.6631 (3)	0.09939 (13)	0.0328 (4)	
H12	1.063405	0.666462	0.038948	0.039*	
C13	1.2868 (3)	0.7305 (3)	0.13960 (13)	0.0296 (4)	
C14	1.3374 (3)	0.7275 (2)	0.22748 (13)	0.0294 (4)	
H14	1.468043	0.775194	0.254682	0.035*	
C15	1.1936 (3)	0.6535 (2)	0.27505 (13)	0.0267 (4)	
C16	0.3685 (3)	0.1376 (3)	0.41071 (13)	0.0297 (4)	
H16A	0.497966	0.120700	0.443893	0.036*	
H16B	0.293516	0.024451	0.390173	0.036*	
C17	0.2378 (3)	0.2424 (3)	0.47210 (13)	0.0324 (4)	
H17A	0.307185	0.358366	0.490295	0.039*	
H17B	0.102609	0.251541	0.441251	0.039*	
C18	0.2103 (3)	0.1586 (3)	0.54900 (14)	0.0337 (5)	

### Atomic displacement parameters (Å^2^)

**Table:**

	*U*^11^	*U*^22^	*U*^33^	*U*^12^	*U*^13^	*U*^23^
Cl1	0.0318 (3)	0.0578 (3)	0.0310 (3)	−0.0053 (2)	0.0060 (2)	0.0108 (2)
Cl2	0.0310 (3)	0.0415 (3)	0.0244 (3)	−0.00393 (19)	−0.00445 (18)	0.0084 (2)
S1	0.0251 (3)	0.0468 (3)	0.0203 (2)	−0.0039 (2)	0.00083 (17)	0.0050 (2)
O1	0.0377 (8)	0.0475 (9)	0.0219 (7)	−0.0087 (7)	−0.0026 (6)	0.0067 (6)
N1	0.0274 (8)	0.0318 (8)	0.0216 (8)	−0.0019 (6)	0.0014 (6)	0.0051 (6)
N2	0.0523 (13)	0.0529 (12)	0.0393 (12)	0.0016 (10)	0.0169 (9)	0.0153 (10)
C1	0.0224 (9)	0.0321 (10)	0.0250 (10)	0.0021 (7)	0.0026 (7)	0.0029 (8)
C2	0.0276 (10)	0.0446 (12)	0.0259 (10)	0.0036 (8)	0.0010 (8)	0.0035 (9)
C3	0.0271 (10)	0.0460 (12)	0.0299 (11)	−0.0001 (9)	−0.0024 (8)	−0.0003 (9)
C4	0.0260 (10)	0.0379 (11)	0.0371 (12)	−0.0031 (8)	0.0017 (8)	0.0006 (9)
C5	0.0308 (10)	0.0342 (10)	0.0307 (11)	−0.0018 (8)	0.0056 (8)	0.0041 (8)
C6	0.0255 (9)	0.0279 (9)	0.0256 (10)	0.0014 (7)	0.0010 (7)	0.0016 (8)
C7	0.0279 (10)	0.0325 (10)	0.0228 (10)	0.0007 (8)	0.0013 (7)	0.0037 (8)
C8	0.0256 (9)	0.0308 (9)	0.0220 (9)	0.0009 (7)	0.0015 (7)	0.0037 (7)
C9	0.0286 (10)	0.0327 (10)	0.0220 (9)	0.0005 (8)	0.0003 (7)	0.0037 (8)
C10	0.0260 (10)	0.0292 (9)	0.0253 (10)	0.0010 (7)	0.0016 (7)	0.0047 (8)
C11	0.0297 (10)	0.0418 (11)	0.0261 (10)	−0.0044 (8)	−0.0028 (8)	0.0071 (9)
C12	0.0326 (11)	0.0418 (11)	0.0235 (10)	−0.0020 (9)	−0.0010 (8)	0.0091 (8)
C13	0.0261 (10)	0.0349 (10)	0.0284 (10)	0.0005 (8)	0.0056 (8)	0.0067 (8)
C14	0.0241 (9)	0.0344 (10)	0.0290 (11)	0.0010 (8)	0.0006 (8)	0.0045 (8)
C15	0.0272 (10)	0.0291 (9)	0.0232 (9)	0.0019 (7)	−0.0012 (7)	0.0041 (7)
C16	0.0317 (10)	0.0331 (10)	0.0254 (10)	0.0003 (8)	0.0036 (8)	0.0095 (8)
C17	0.0358 (11)	0.0342 (10)	0.0273 (11)	−0.0006 (8)	0.0052 (8)	0.0063 (8)
C18	0.0335 (11)	0.0378 (11)	0.0296 (11)	−0.0002 (8)	0.0083 (8)	0.0033 (9)

### Geometric parameters (Å, º)

**Table:**

Cl1—C13	1.741 (2)	C7—C8	1.501 (3)
Cl2—C15	1.742 (2)	C8—C9	1.353 (3)
S1—C8	1.7407 (19)	C9—C10	1.452 (3)
S1—C1	1.7411 (19)	C9—H9	0.9500
O1—C7	1.226 (2)	C10—C11	1.407 (3)
N1—C7	1.375 (3)	C10—C15	1.415 (3)
N1—C6	1.421 (2)	C11—C12	1.380 (3)
N1—C16	1.469 (2)	C11—H11	0.9500
N2—C18	1.144 (3)	C12—C13	1.383 (3)
C1—C6	1.397 (3)	C12—H12	0.9500
C1—C2	1.397 (3)	C13—C14	1.381 (3)
C2—C3	1.380 (3)	C14—C15	1.384 (3)
C2—H2	0.9500	C14—H14	0.9500
C3—C4	1.384 (3)	C16—C17	1.538 (3)
C3—H3	0.9500	C16—H16A	0.9900
C4—C5	1.378 (3)	C16—H16B	0.9900
C4—H4	0.9500	C17—C18	1.462 (3)
C5—C6	1.395 (3)	C17—H17A	0.9900
C5—H5	0.9500	C17—H17B	0.9900
			
Cl2···C18^i^	3.649 (2)	N2···H16A^ix^	2.81
Cl2···C7^ii^	3.520 (2)	C2···C11^vi^	3.569 (3)
Cl2···O1^i^	3.0269 (15)	C4···C12^x^	3.577 (3)
Cl1···H2^iii^	3.00	C4···C8^vi^	3.490 (3)
Cl1···H4^iv^	2.94	C5···C14^x^	3.557 (3)
Cl2···H17A^ii^	3.06	C5···C17	3.352 (3)
Cl2···H9	2.51	C8···C4^ii^	3.490 (3)
Cl2···H16B^v^	2.96	C9···C18^vii^	3.497 (3)
S1···N1	3.1168 (17)	C11···C2^ii^	3.569 (3)
S1···C3^ii^	3.598 (2)	C12···C4^v^	3.577 (3)
S1···C4^ii^	3.510 (2)	C14···C5^v^	3.557 (3)
S1···C11	3.162 (2)	C17···C5	3.352 (3)
S1···C14^vi^	3.578 (2)	C18···C9^vii^	3.497 (3)
S1···H11	2.47	C5···H16B	2.53
O1···C17	3.210 (2)	C5···H17B	2.86
O1···Cl2^i^	3.0269 (15)	C8···H11	2.94
O1···C17^vii^	3.336 (3)	C16···H5	2.48
O1···H17A	2.79	C17···H5	2.79
O1···H9	2.24	C18···H9^vii^	2.98
O1···H16A	2.29	H2···H12^xi^	2.49
O1···H17A^vii^	2.45	H5···H16B	2.03
N2···C5^viii^	3.282 (3)	H5···H17B	2.26
N2···H5^viii^	2.43		
			
C8—S1—C1	103.69 (9)	C11—C10—C15	115.36 (18)
C7—N1—C6	126.27 (16)	C11—C10—C9	125.21 (18)
C7—N1—C16	114.86 (16)	C15—C10—C9	119.43 (18)
C6—N1—C16	118.72 (16)	C12—C11—C10	122.73 (19)
C6—C1—C2	120.06 (18)	C12—C11—H11	118.6
C6—C1—S1	123.84 (15)	C10—C11—H11	118.6
C2—C1—S1	116.06 (15)	C11—C12—C13	119.12 (19)
C3—C2—C1	120.8 (2)	C11—C12—H12	120.4
C3—C2—H2	119.6	C13—C12—H12	120.4
C1—C2—H2	119.6	C14—C13—C12	121.32 (18)
C2—C3—C4	119.0 (2)	C14—C13—Cl1	119.49 (15)
C2—C3—H3	120.5	C12—C13—Cl1	119.19 (16)
C4—C3—H3	120.5	C13—C14—C15	118.53 (18)
C5—C4—C3	120.8 (2)	C13—C14—H14	120.7
C5—C4—H4	119.6	C15—C14—H14	120.7
C3—C4—H4	119.6	C14—C15—C10	122.93 (18)
C4—C5—C6	121.0 (2)	C14—C15—Cl2	116.72 (15)
C4—C5—H5	119.5	C10—C15—Cl2	120.34 (15)
C6—C5—H5	119.5	N1—C16—C17	112.76 (16)
C5—C6—C1	118.32 (18)	N1—C16—H16A	109.0
C5—C6—N1	120.22 (18)	C17—C16—H16A	109.0
C1—C6—N1	121.46 (17)	N1—C16—H16B	109.0
O1—C7—N1	119.47 (18)	C17—C16—H16B	109.0
O1—C7—C8	119.89 (18)	H16A—C16—H16B	107.8
N1—C7—C8	120.59 (17)	C18—C17—C16	108.89 (17)
C9—C8—C7	114.77 (17)	C18—C17—H17A	109.9
C9—C8—S1	123.67 (15)	C16—C17—H17A	109.9
C7—C8—S1	121.12 (14)	C18—C17—H17B	109.9
C8—C9—C10	132.12 (19)	C16—C17—H17B	109.9
C8—C9—H9	113.9	H17A—C17—H17B	108.3
C10—C9—H9	113.9	N2—C18—C17	176.4 (2)
			
C8—S1—C1—C6	−13.00 (19)	N1—C7—C8—S1	−3.4 (3)
C8—S1—C1—C2	168.95 (15)	C1—S1—C8—C9	−173.92 (17)
C6—C1—C2—C3	−0.4 (3)	C1—S1—C8—C7	14.09 (18)
S1—C1—C2—C3	177.68 (17)	C7—C8—C9—C10	173.6 (2)
C1—C2—C3—C4	1.0 (3)	S1—C8—C9—C10	1.1 (3)
C2—C3—C4—C5	−0.4 (3)	C8—C9—C10—C11	9.6 (4)
C3—C4—C5—C6	−0.7 (3)	C8—C9—C10—C15	−169.6 (2)
C4—C5—C6—C1	1.3 (3)	C15—C10—C11—C12	1.6 (3)
C4—C5—C6—N1	−178.01 (19)	C9—C10—C11—C12	−177.6 (2)
C2—C1—C6—C5	−0.7 (3)	C10—C11—C12—C13	−0.9 (3)
S1—C1—C6—C5	−178.69 (15)	C11—C12—C13—C14	−0.3 (3)
C2—C1—C6—N1	178.59 (18)	C11—C12—C13—Cl1	179.24 (17)
S1—C1—C6—N1	0.6 (3)	C12—C13—C14—C15	0.6 (3)
C7—N1—C6—C5	−165.80 (19)	Cl1—C13—C14—C15	−178.93 (15)
C16—N1—C6—C5	9.4 (3)	C13—C14—C15—C10	0.2 (3)
C7—N1—C6—C1	14.9 (3)	C13—C14—C15—Cl2	179.63 (15)
C16—N1—C6—C1	−169.86 (18)	C11—C10—C15—C14	−1.3 (3)
C6—N1—C7—O1	169.33 (18)	C9—C10—C15—C14	177.97 (18)
C16—N1—C7—O1	−6.1 (3)	C11—C10—C15—Cl2	179.34 (15)
C6—N1—C7—C8	−13.1 (3)	C9—C10—C15—Cl2	−1.4 (3)
C16—N1—C7—C8	171.47 (17)	C7—N1—C16—C17	88.6 (2)
O1—C7—C8—C9	1.4 (3)	C6—N1—C16—C17	−87.2 (2)
N1—C7—C8—C9	−176.09 (18)	N1—C16—C17—C18	−175.75 (17)
O1—C7—C8—S1	174.09 (16)		

Symmetry codes: (i) −*x*+2, −*y*+1, −*z*+1; (ii) *x*+1, *y*, *z*; (iii) −*x*+2, −*y*+1, −*z*; (iv) *x*+2, *y*+1, *z*; (v) *x*+1, *y*+1, *z*; (vi) *x*−1, *y*, *z*; (vii) −*x*+1, −*y*+1, −*z*+1; (viii) −*x*, −*y*, −*z*+1; (ix) −*x*+1, −*y*, −*z*+1; (x) *x*−1, *y*−1, *z*; (xi) −*x*+1, −*y*+1, −*z*.

### Hydrogen-bond geometry (Å, º)

**Table:**

*D*—H···*A*	*D*—H	H···*A*	*D*···*A*	*D*—H···*A*
C5—H5···N2^viii^	0.95	2.43	3.282 (3)	149
C17—H17*A*···O1^vii^	0.99	2.45	3.337 (3)	149

Symmetry codes: (vii) −*x*+1, −*y*+1, −*z*+1; (viii) −*x*, −*y*, −*z*+1.
